# The duration and body position during tongue-kissing among heterosexual men and women

**DOI:** 10.3389/fpubh.2022.934962

**Published:** 2022-12-22

**Authors:** Julien Tran, Christopher K. Fairley, Jason J. Ong, Catriona S. Bradshaw, Ei T. Aung, Kate Maddaford, Marcus Y. Chen, Jane S. Hocking, Eric P. F. Chow

**Affiliations:** ^1^Melbourne Sexual Health Centre, Alfred Health, Melbourne, VIC, Australia; ^2^Central Clinical School, Faculty of Medicine, Nursing and Health Sciences, Monash University, Melbourne, VIC, Australia; ^3^Centre for Epidemiology and Biostatistics, Melbourne School of Population and Global Health, The University of Melbourne, Melbourne, VIC, Australia

**Keywords:** tongue kiss, heterosexual, sex, saliva, sexually transmitted infection

## Abstract

**Background:**

Emerging data suggest tongue-kissing may transmit gonorrhea. We aim to examine the duration or body position of heterosexual men and women during tongue-kissing (henceforth, known as kissing).

**Methods:**

A cross-sectional survey among heterosexual men and women attending the Melbourne Sexual Health Centre in Australia between May 2019 and March 2020 collected data on the duration and body position (i.e., on top of or lying down underneath) of their most recent kissing partner in the past 3 months. Univariable and multivariable linear regressions were performed to examine the association between gender and kissing duration.

**Results:**

Of 2,866 individuals, 93.6% (*n* = 2,683) had at least one kissing partner in the past 3 months, which included 1,342 (50.1%) men and 1,341 (49.9%) women, and 87.2% (*n* = 2,339) had sex with their opposite-gender kissing partner. The adjusted mean duration of kissing with the most recent opposite-gender kissing partner did not differ between men and women (12.2 vs. 11.5 min, *p* = 0.170). More men were on top of their most recent opposite-gender kissing partner compared to women (87.9 vs. 82.9%, *p* < 0.001). Men reported a longer kissing duration than women when they were on top of the opposite-gender kissing partner (8.3 vs. 7.4 min, *p* = 0.006). More women had same-gender kissing partners than men (9.6 vs. 2.8%, *p* < 0.001).

**Conclusion:**

Men spending longer than women on top of their opposite-gender kissing partner suggests a potential alternative explanation for oropharyngeal gonorrhea being seen more commonly in women. Further research should investigate whether body positioning and duration of kissing influence the risk of gonorrhea transmission.

## Introduction

Gonorrhea notifications have been rising substantially in many countries in the 2010s, especially among gay, bisexual, and other men who have sex with men (MSM) (1–3). Condomless anal sex has also been increasing among MSM since the introduction of pre-exposure prophylaxis (PrEP) to prevent Human Immunodeficiency Virus (HIV) (4–7). Since the late 2010s, gonorrhea notifications have also been rising among heterosexuals in many high-income countries (8–10), despite no reductions in condom use (11, 12). This increase in gonorrhea notifications has raised questions about how *Neisseria gonorrhoeae* is transmitted (13–15).

Several researchers have questioned the generally accepted transmission routes of gonorrhea and proposed that tongue-kissing (with or without sex) may also transmit gonorrhea (16). Supporting this proposition are observations that gonorrhea can frequently be cultured in the saliva of individuals with oropharyngeal gonorrhea (17, 18) and data from several epidemiological studies (19, 20). If kissing transmits a substantial proportion of oropharyngeal gonorrhea, then one would assume that the prevalence of oropharyngeal gonorrhea would be similar in heterosexual men and women. However, some studies have shown that oropharyngeal gonorrhea is more common in heterosexual women than heterosexual men (21–24). This observation has been attributed to fellatio being more efficient in transmitting gonorrhea from the penis to the oropharynx than cunnilingus from the vulva to the oropharynx (21–25). However, a past study has shown that condomless fellatio is not an independent risk factor for oropharyngeal gonorrhea in female sex workers (26), suggesting the difference in the prevalence of oropharyngeal gonorrhea between men and women may be due to other factors.

We hypothesize that oropharyngeal gonorrhea is more common in heterosexual women than heterosexual men because women are likely to be underneath their male partner during sex (27). Only a few studies have examined kissing among heterosexual men and women (28–34). For instance, one study found that relational or sexual motives (e.g., pleasure, affection) were the most common motives for kissing (33). Charleson et al. (32) found that on average, younger men compared to older men had more kissing-only partners, and that kissing-only partners varied across regions of birth (32). A US study of 738 male and female university students found that students who have never been kissed were more likely to Asian-American, and less likely to be in a relationship, were less extraverted, and drank less alcohol (30). These studies focused on motivations of kissing, and trends and associated factors in kissing, however, none have described the body position of men or women during kissing. Therefore, we aimed to examine the kissing practices of heterosexual men and women, particularly the duration of kissing and the body position while kissing, which may provide a better understanding of how oropharyngeal gonorrhea is transmitted.

## Methods

### Study setting and population

The “Kissing And Sexual Practices” (KASP) was a cross-sectional survey conducted at the Melbourne Sexual Health Centre (MSHC) in Melbourne (Victoria, Australia) between 1-May 2019 and 13-March 2020. The KASP survey to examine whether kissing and other sexual practices plays a role in transmission of sexually transmissible infections (STIs). We planned to run the KASP survey for 12 months but stopped early in March 2020 due to the COVID-19 pandemic, as the kissing practices and clinic attendees might have changed during the COVID-19 pandemic (35, 36). MSHC is a major public sexual health clinic and provided about 52,000 consultations in 2019 (37). Upon arrival, clients are asked to register their clinic visit and complete questions about their demographic characteristics (e.g., age, country of birth), and sexual history (e.g., number of partners, condom use) as part of routine care, using computer-assisted self-interviewing (CASI) (38). At the end of these routine questions, all men and women who reported sex with opposite-gender partners only in the past 12 months and aged 16 years or older were invited *via* CASI to participate in the KASP survey. The survey included two questions about their kissing practices. Participation was voluntary, and no payment was given for survey completion.

Individuals who agreed to participate in the KASP survey provided consent by clicking “Yes,” and a “No” option was also offered for those who did not want to participate on the first page of the survey. If the participants had completed the survey more than once, only their first completed KASP response was included. Sex workers and incomplete surveys were excluded. Surveys were considered incomplete if they did not proceed to the survey's last question. This study was approved by the Alfred Hospital Ethics Committee, Melbourne, Australia (647/17).

### Measurement

The KASP survey included two questions about kissing practices. Kissing was defined as tongue-kissing with another individual. Participants were first asked if they had kissed a man and/or woman in the past 3 months and whether they had sex with the person whom they kissed most recently. Sex was defined as any type of sexual contact (e.g., oral, vaginal, and/or anal sex). Participants' responses to these questions were grouped into two categories: kissing-with-sex and kissing-without-sex. The number of ‘any kissing partners’ was defined as the sum of kissing-with-sex and kissing-without-sex partners. Participants were then asked to self-report an estimate of the duration (in minutes) of their most recent tongue kiss with a man and/or woman and for the duration of kissing when they were on top of and when they were lying down underneath their most recent kissing partner, respectively (Supplementary Figure 1). Routinely collected data on the participants' demographic characteristics (i.e., age, country of birth) were extracted from the clinic's secured electronic database by a data analyst. Data was accessed and analyzed by the research team only.

### Statistical analysis

In this analysis, we excluded individuals who reported kissing duration to be more than 60 min (e.g., some individuals reported their tongue-kissing time was 300 min). It was likely that the participants might have either misunderstood the question or had typing mistakes *via* CASI. We created six categories for region of birth based on continent: (1) Australia and Oceania; (2) Asia; (3) Europe; (4) Middle East or Africa; (5) North America; (6) South America or Caribbean (32). Mean and standard deviation (SD) were calculated for continuous variables such as age and duration of kissing. In contrast, frequency and proportion were calculated for categorical variables, such as region of birth and the proportion of tongue kissing for opposite-gender and same-gender partners. A *t*-test was used to compare the mean difference for continuous variables between groups, while a Chi-square test was used to compare the difference for categorical variables between groups. Univariable and multivariable linear regression analyses were conducted to examine the association between demographic factors and the duration of kissing with the most recent opposite-gender kissing partners. We examined demographic factors such as gender, age, region of birth, and whether individuals had a regular sex partner and/or casual sex partners. Variables with a *p* < 0.20 in the univariable analysis were considered as confounding factors and were included in the multivariable analysis (39). The adjusted marginal means and the corresponding 95% confidence intervals (CI) for each demographic factor was calculated. We also performed univariable and multivariable linear regression analyses to examine the duration of kissing while (a) on top of, and (b) lying down underneath their most recent opposite-gender kissing partners, separately. All data analyses were performed using Stata (version 17, Stata Corp., College Station, TX, USA).

## Results

### Study population

Between May 2019 and March 2020, 14,130 heterosexual men and women (6,805 men and 7,325 women) who completed the routine questionnaire on CASI were invited to participate in the KASP survey. Of those, 3,282 (23.2%) consented to participate, including 1,521 men and 1,761 women. Individuals who consented to participate were significantly older than those who did not consent to participate in the study (mean = 29.7 vs. 29.2 years, *p* = 0.002).

Of the 1,521 men, we excluded 25 men who reported having had sex with other men that they had not reported on CASI. Of the 1,761 women, we excluded 300 women who had sex (i.e., more than kissing) with other women. We further excluded 91 responses, including 58 who reported the duration of kissing was more than 60 min and 33 who had inconsistent data (i.e., the total duration of any kissing was shorter than the duration of kissing while they were on top of or lying down underneath their most recent kissing partner). After these exclusions, 2,866 individuals remained, of whom 93.6% (*n* = 2,683) had at least one kissing partner in the past 3 months.

We included the 2,683 individuals who had at least one kissing partner in the final analysis: 1,342 (50.1%) men and 1,341 (49.9%) women. Their ages ranged from 16 to 81 years, with a mean age of 29.4 (SD = 8.4). Most men and women were born in Australia or Oceania (37.4%, *n* = 1,004), followed by Europe (26.3%, *n* = 705), Asia (12.5%, *n* = 336), South America or Caribbean (9.0%, *n* = 240), North America (6.9%, *n* = 185), and Middle East or Africa (2.5%, *n* = 68). The mean number of casual sex partners in the past 3 months was significantly greater among men (mean = 2.8, SD = 3.5) than women (mean = 2.2, SD = 2.2) (*p* < 0.001), while the proportion of men who had casual sex partners was similar to women (*p* = 0.460) (Table 1). The proportion of men who had a regular sex partner was similar to women (*p* = 0.287).

**Table 1:** Demographic characteristics and duration of kissing among 2,683 heterosexual men and women in the past 3 months.

	**All (*N* = 2,683)**	**Men (*N* = 1,342)**	**Women (*N* = 1,341)**	***P*** -value^*^
**Demographic characteristics**				
Age, mean ± SD	29.4 ± 8.4	31.5 ± 9.8	27.4 ± 6.1	< 0.001
**Region of birth**, ***n*** **(%)**				< 0.001
Australia or Oceania	1,004 (37.4%)	605 (45.1%)	399 (29.8%)	
Asia	336 (12.5%)	152 (11.3%)	184 (13.7%)	
Europe	705 (26.3%)	328 (24.4%)	377 (28.1%)	
Middle East or Africa	68 (2.5%)	38 (2.8%)	30 (2.2%)	
North America	185 (6.9%)	66 (4.9%)	119 (8.9%)	
South America or Caribbean	240 (9.0%)	77 (5.7%)	163 (12.2%)	
Unknown/missing	145 (5.45)	76 (5.7%)	69 (5.2%)	
**Regular sex partner**				0.287
Yes	1,198 (44.7%)	619 (46.1%)	579 (43.2%)	
No	1,412 (52.6)	689 (51.3%)	723 (53.9%)	
Unknown/missing	73 (2.7%)	34 (2.5%)	39 (2.9%)	
**Casual sex partners**				0.460
Yes	2,021 (75.3%)	1,012 (75.4%)	1,009 (75.2%)	
No	353 (13.2%)	168 (12.5%)	185 (13.8%)	
Unknown/missing	309 (11.5%)	162 (12.1%)	147 (11.0%)	
Number of casual sex partners, mean ± SD	2.4 ± 2.9	2.7 ± 3.5	2.2 ± 2.2	< 0.001
**Opposite-gender kissing partners in the past 3 months**				
**Most recent opposite-gender kissing partner**				
Number of individuals who had sex with their most recent kissing partner (i.e., kissing-with-sex partner), *n* (%)	2,339 (87.2%)	1,182 (88.1%)	1,157 (86.3%)	0.164
Duration of kissing-with-sex partner (minutes), median (IQR)	5 (5–15)	7 (3–20)	5 (2–15)	
Number of individuals who did not have sex with their most recent kissing partner (i.e., kissing-without-sex partner), *n* (%)	344 (12.8%)	160 (11.9%)	184 (13.7%)	0.152
Duration of kissing-without-sex (minutes), median (IQR)	5 (2–10)	5 (2–12.5)	5 (2–10)	
**Same-gender kissing partners in past 3 months** ^**^				
Number of individuals who had same-gender kissing partners, *n* (%)	169 (6.2%)	40 (2.8%)	129 (9.6%)	< 0.001

* The two-sample independent t-test was used to compare the mean age between men and women. The chi-square test was used to compare the region of birth and the proportion of kissing partners between men and women.

** Individuals reported kissing-without-sex only when they kissed their most recent same-gender partner in the past 3 months.

SD, standard deviation; IQR, interquartile range.

### Kissing duration

The median duration of kissing with the most recent opposite-gender kissing partner was 5 min (Interquartile Range; IQR: 3–15), respectively. Overall, 87.2% (*n* = 2,339) had sex with their most recent opposite-gender kissing partner; however, there was no differences between men (88.1%, *n* = 1,182) and women (86.3%, *n* = 1,157) (*p* = 0.164). The median duration of kissing-with-sex (median = 5 min, IQR: 3–15) and kissing-without-sex (median = 5 min, IQR: 2–10) were similar.

The univariable linear regression showed that gender, region of birth, having a regular partner, and having a casual partner were associated with the duration of kissing; however, age was not associated with the duration of kissing (Table 2). The adjusted mean duration of kissing was 12.22 min among men (95% CI: 11.50–12.96) and 11.49 min (95% CI: 10.77 to 12.22) among women; however, the difference was not statistically significant after adjusting other demographic characteristics (adjusted regression coefficient = 0.73; 95% CI: −0.31 to 1.76, *p* = 0.170). The adjusted mean duration of kissing among individuals born in Australia or Oceania was, on average, significantly longer compared to individuals born in Asia, Europe, South America or the Caribbean. There were no significant differences in mean duration of kissing between individuals born in Australia or Oceania and individuals born in the Middle East or Africa, and North America. The adjusted mean duration of kissing among individuals who had a regular partner was on average 1.42 (95% CI: 0.28 to 2.56, *p* = 0.015) minutes shorter compared to those who did not have a regular partner (i.e., 11.05 vs. 12.47 min, respectively). However, the adjusted mean duration of kissing among individuals who had a casual partner was on average 3.19 (95% CI: 1.58 to 4.79, *p* < 0.001) minutes longer compared to those who did not have a casual partner (i.e., 12.61 vs. 9.43 min, respectively).

**Table 2:** Univariable and multivariable linear regression analyses of the association between demographic factors and the duration of kissing among 2,683 heterosexual men and women who had opposite-gender kissing partners in the past 3 months.

**Predictors**	**Kissing duration, median (IQR)**	**Crude regression coefficient (95% CI)**	**Unadjusted mean (95% CI)**	***P*** **-value**	**Adjusted regression coefficient (95% CI)**	**Adjusted mean (95% CI)**	***P*** **-value**
**Gender**							
Female	5 (2–15)	1	11.20 (10.46, 11.93)	Ref	1	11.49 (10.77, 12.22)	Ref
Male	5 (3–20)	1.31 (0.26, 2.35)	12.51 (11.77,13.24)	0.014	0.73 (−0.31, 1.76)	12.22 (11.49, 12.94)	0.170
**Age (years)**		−0.02 (−0.08, 0.05)		0.607			
**Country of birth**							
Australia or Oceania	10 (5–20)	1	14.5 (13.66, 15.34)	Ref	1	14.46 (13.63, 15.30)	Ref
Asia	3 (2–10)	−7.66 (−9.33, −5.99)	6.84 (5.39, 8.29)	< 0.001	−7.26 (−8.94, −5.57)	7.21 (5.75, 8.66)	< 0.001
Europe	5 (2–11)	−3.76 (−5.06, −2.46)	10.74 (9.74, 11.74)	< 0.001	−3.92 (−5.22, −2.61)	10.54 (9.55, 11.54)	< 0.001
Middle East or Africa	5 (3–10)	−3.51 (−6.83, −0.19)	10.99 (7.77, 14.20)	0.038	−3.21 (−6.52, 0.09)	11.25 (8.05, 14.45)	0.057
North America	10 (5–20)	0.75 (−1.37, 2.88)	15.25 (13.31, 17.20)	0.486	0.55 (−1.58, 2.67)	15.01 (13.06, 16.96)	0.614
South America or Caribbean	5 (2–10)	−5.89 (−7.79, −3.98)	8.61 (6.90, 10.32)	< 0.001	−5.51 (−7.42, −3.59)	8.95 (7.24, 10.66)	< 0.001
Unknown	9 (5–15)	−2.51 (−4.86, −0.15)	11.99 (9.79, 14.19)	0.037	−2.49 (−4.82, −0.15)	11.98 (9.79, 14.16)	0.037
**Regular sex partners**							
No	8 (3–20)	1	13.18 (12.47, 13.9)	Ref	1	12.47 (11.73, 13.21)	Ref
Yes	5 (2–15)	−2.83 (−3.89, −1.78)	10.35 (9.58, 11.13)	< 0.001	−1.42 (−2.56, −0.28)	11.05 (10.25, 11.85)	0.015
Unknown	3 (2–10)	−2.33 (−5.56, 0.89)	10.85 (7.70, 14.00)	0.157	0.67 (−2.67, 4.01)	13.14 (9.93, 16.35)	0.695
**Casual sex partners**							
No	5 (2–10)	1	9.08 (7.65, 10.50)	Ref	1	9.43 (7.97, 10.89)	Ref
Yes	7 (3–20)	3.74 (2.19, 5.29)	12.82 (12.23, 13.42)	< 0.001	3.19 (1.58, 4.79)	12.61 (12.01, 13.22)	< 0.001
Unknown	5 (2–10)	−0.39 (−2.48, 1.70)	8.69 (7.16, 10.21)	0.712	0.20 (−1.91, 2.31)	9.63 (8.02, 11.24)	0.853

CI, confidence interval.

IQR, interquartile range.

Some men and women also kissed same-gender partners. More women [9.6% (129/1,341)] reported kissing same-gender partners than men [2.8% (40/1,342)] (*p* < 0.001). The duration of kissing of the 40 men who kissed another man [mean 3.70 min (SD = 4.00)] was not significantly different from the 129 women who kissed another woman [mean 4.70 min (SD = 7.30)] (*p* = 0.388). The duration of kissing-without-sex was longer with opposite-gender partners than with same-gender partners (10.50 vs. 4.30 min, *p* < 0.001).

### Body position during kissing

A significantly greater proportion of men compared to women were on top of their most recent opposite-gender kissing partner [87.9% (1,180/1,342) vs. 82.9% (1,111/1,341), *p* < 0.001]. Of the 2,291 men and women who were on top, the adjusted mean duration of kissing while on top was 8.27 (95% CI: 7.83 to 8.70) minutes among men and 7.40 min among women (95% CI: 6.95 to 7.84), and the difference between men and women was statistically significant after adjusting other demographic characteristics (adjusted regression coefficient = 0.86; 95% CI: 0.24 to 1.50, *p* = 0.006) (Table 3).

**Table 3:** Univariable and multivariable linear regression analyses of the association between demographic factors and the duration of kissing while on top of partner among 2,291 heterosexual men and women who had opposite-gender kissing partners in the past 3 months.

**Predictors**	**Kissing duration, median (IQR)**	**Crude regression coefficient (95% CI)**	**Unadjusted mean (95% CI)**	***P*** **-value**	**Adjusted regression coefficient (95% CI)**	**Adjusted means (95% CI)**	***P*** **-value**
**Gender**							
Female	5 (2–10)	1	7.23 (6.78, 7.68)	Ref	1	7.40 (6.95, 7.84)	Ref
Male	5 (3–10)	1.19 (0.56, 1.82)	8.41 (7.98, 8.86)	< 0.001	0.86 (0.24, 1.50)	8.27 (7.83, 8.70)	0.006
**Age (years)**		−0.01 (−0.04, 0.04)		0.894			
**Country of birth**							
Australia or Oceania	5 (3–10)	1	9.13 (8.63, 9.63)	Ref	1	9.05 (8.54, 9.55)	Ref
Asia	3 (2–5)	−4.21 (−5.24, −3.18)	4.92 (4.02, 5.82)	< 0.001	−3.93 (−4.96, −2.89)	5.12 (4.22, 6.02)	< 0.001
Europe	5 (3–10)	−1.78 (−2.57, −0.99)	7.35 (6.74, 7.95)	< 0.001	−1.76 (−2.55, −0.97)	7.28 (6.68, 7.89)	< 0.001
Middle East or Africa	5 (3–10)	−0.92 (−2.87, 1.03)	8.21 (6.33, 10.09)	0.354	−0.76 (−2.71, 1.19)	8.29 (6.41, 10.17)	0.446
North America	5 (3–10)	−0.08 (−1.40, 1.23)	9.05 (7.83, 10.26)	0.900	−0.02 (−1.34, 1.30)	9.02 (7.81, 10.24)	0.971
South America or Caribbean	5 (2–10)	−2.39 (−3.52, −1.26)	6.74 (5.73, 7.76)	< 0.001	−2.04 (−3.19, −0.90)	7.00 (5.98, 8.02)	< 0.001
Unknown	5 (3–10)	−1.25 (−2.68, 0.17)	7.88 (6.54, 9.21)	0.085	−1.16 (2.58, 0.26)	7.89 (6.56, 9.22)	0.110
**Regular sex partners**							
No	5 (3–10)	1	8.44 (8.00, 8.87)	Ref	1	8.15 (7.69, 8.61)	Ref
Yes	5 (2–10)	−1.24 (−1.88, −0.60)	7.19 (6.73, 7.66)	< 0.001	−0.69 (−1.38, 0.01)	7.46 (6.98, 7.94)	0.052
Unknown	5 (1.5–10)	−0.72 (−2.77, 1.33)	7.71 (5.71, 9.72)	0.491	0.50 (−1.61, 2.61)	8.65 (6.61, 10.69)	0.640
**Casual sex partners**							
No	5 (2–10)	1	6.86 (6.00, 7.72)	Ref	1	7.11 (6.22, 7.99)	Ref
Yes	5 (3–10)	1.35 (0.42, 2.29)	8.21 (7.85, 8.58)	0.005	1.01 (0.03, 1.99)	8.11 (7.75, 8.48)	0.044
Unknown	5 (27)	−0.36 (−1.64, 0.91)	6.50 (5.56, 7.44)	0.576	−0.22 (−1.50, 1.07)	6.89 (5.90, 7.88)	0.740

CI, confidence interval.

IQR, interquartile range.

A significantly smaller proportion of men compared to women reported lying underneath their most recent opposite-gender kissing partners [80.0% (1,073/1,341) vs. 84.0% (1,126/1,341), *p* < 0.001]. Of the 2,199 men and women who were lying underneath their partner, the adjusted mean duration of kissing while lying underneath their partner was 7.53 (95% CI: 7.07 to 7.99) min among men and 8.34 (95% CI: 7.89 to 8.79) min among women, and the difference between men and women was statistically significant after adjusting other demographic characteristics (adjusted regression coefficient = −0.81; 95% CI: −1.45 to −0.16, *p* = 0.014) (Table 4).

**Table 4:** Univariable and multivariable linear regression analyses of the association between demographic factors and the duration of kissing while lying down underneath partner among 2,199 heterosexual men and women who had opposite-gender kissing partners in the past 3 months.

**Predictors**	**Kissing duration, median (IQR)**	**Crude regression coefficient (95% CI)**	**Unadjusted Mean (95% CI)**	***P*** **-value**	**Adjusted regression coefficient (95% CI)**	**Adjusted mean (95% CI)**	***P*** **-value**
**Gender**							
Female	5 (3–10)	1	8.21 (7.76, 8.66)	Ref	1	8.34 (7.89, 8.79)	Ref
Male	5 (2–10)	−0.54 (−1.19, −0.10)	7.67 (7.21, 8.13)	0.097	−0.81 (−1.45, −0.16)	7.53 (7.07, 7.99)	0.014
**Age (years)**		−0.1 (−0.05, −0.03)		0.507			
**Country of birth**							
Australia or Oceania	5 (3–10)	1	8.96 (8.44, 9.48)	Ref	1	9.06 (8.54, 9.58)	Ref
Asia	3 (1–15)	−3.96 (−5.02, −2.89)	5.00 (4.07, 5.93)	< 0.001	−3.87 (−4.94, −2.80)	5.19 (4.25, 6.12)	< 0.001
Europe	5 (3–10)	−1.25 (−2.06, −0.45)	7.71 (7.09, 8.32)	0.002	−1.52 (−2.33 −0.71)	7.54 (6.92, 8.15)	< 0.001
Middle East or Africa	5 (2–9.5)	−1.51 (−3.57, 0.54)	7.45 (5.46, 9.44)	0.149	−1.47 (−3.51, 0.57)	7.59 (5.61, 9.57)	0.158
North America	5 (3–15)	0.25 (−1.06, 1.57)	9.21 (8.01, 10.42)	0.708	−0.14 (−1.46, 1.18)	8.92 (7.71, 10.13)	0.838
South America or Caribbean	5 (2–10)	−1.46 (−2.62, −0.30)	7.50 (6.46, 8.54)	0.014	−1.60 (−2.78, −0.43)	7.46 (6.42, 8.50)	0.007
Unknown	5 (3–10)	−1.24 (−2.70, 0.22)	7.72 (6.36, 9.09)	0.096	−1.22 (−2.67, 0.24)	7.84 (6.48, 9.21)	0.101
**Regular sex partners**							
No	5 (3–10)	1	8.71 (8.26, 9.15)	Ref	1	8.48 (8.01, 8.94)	Ref
Yes	5 (2–10)	−1.61 (−2.26, −0.96)	7.09 (6.62, 7.57)	0.001	−1.16 (−1.88, −0.45)	7.31 (6.82, 7.81)	0.001
Unknown	5 (2–12.5)	−0.90 (−3.02, 1.23)	7.81 (5.73, 9.89)	0.407	0.13 (−2.06, 2.32)	8.61 (6.49, 10.72)	0.907
**Casual sex partners**							
No	5 (2–10)	1	7.45 (6.55, 8.34)	Ref	1	7.79 (6.86, 8.72)	Ref
Yes	5 (3–10)	0.81 (0.16, 1.78)	8.26 (7.89, 8.62)	0.101	0.32 (−0.70, 1.34)	8.11 (7.74, 8.48)	0.536
Unknown	5 (2–10)	−2.44 (−2.43, 0.20)	6.33 (5.36, 7.30)	0.080	−0.82 (−2.16, 0.51)	6.96 (5.95, 7.98)	0.226

CI, confidence interval.

IQR, interquartile range.

We undertook a sensitivity analysis focusing on heterosexual men and women with and without same-gender kissing partners in the past three months, and the results were similar (see Supplementary Tables 1–3).

## Discussion

To our knowledge, this is the first study to describe differences in the duration of kissing relative to body position among heterosexual men and women. We found that the kissing practices of heterosexual men and women align with the finding in some studies that oropharyngeal gonorrhea is more common in women than in men (21–25). Specifically, we found that compared to women, more men reported being on top of their partner during kissing, and when men were on top, they kissed for longer in this position. If saliva transmitted *N. gonorrhoeae*, then gravity could favor saliva flow from the person on top to the person underneath and perhaps promote pooling of saliva, thereby prolonging exposure to *N. gonorrhoeae*. Our finding that men are on top more than women is consistent with published data (27). Theoretically, this positioning may cause a greater downward flow of saliva containing viable *N. gonorrhoeae* and potentially explain why women appear to be more commonly infected than men at the oropharynx.

As a sexual and/or non-sexual act, kissing is understudied in the literature, and particularly, kissing as a mode of transmission for infectious diseases. Past research has demonstrated that deep or intimate kissing is a significant risk factor for the transmission of *Neisseria meningitidis* (40–43) and Epstein-Barr Virus (EBV) (44–47). For instance, a study of Australian university students found that compared to individuals who did not engage in intimate kissing in the previous week, individuals who engaged in intimate kissing with more than one person in the previous week had 5.5 times higher odds of increased carriage of *N. meningitidis* (41). A study of Edinburgh University students found that the EBV positivity was significantly higher (79%) among women than men (68%) (*p* < 0.001) and higher among those who had penetrative sexual intercourse (83%) compared to those who had not (64%) (*p* < 0.001) (48). The authors of the Edinburg University study suggested that ‘deep kissing’ during sex can increase EBV dosage and thus, increase the risk of transmission through oral contact (48). Given that deep or intimate kissing (which may involve tongue insertion) is a significant risk factor for increased carriage of EBV and *N*. *meningitidis*, tongue-kissing may also be an important route for transmission of oropharyngeal gonorrhea, even within settings that may not involve sex (49).

Our study found that heterosexual men and women engaged in same-gender kissing in the absence of sex. Most previous kissing studies have focused on opposite-gender kissing partners in heterosexuals (28, 30, 32) or same-gender kissing partners in MSM (50–53). However, our study examined same-gender kissing partners in heterosexuals and found that same-gender kissing occurred in a significant number of heterosexuals in the past 3 months and was more common among heterosexual women than in heterosexual men. Our study also found that among heterosexual individuals, the duration of kissing same-gender partners was significantly shorter than kissing opposite-gender partners. The shorter duration of kissing same-gender partners may have resulted from alcohol and drug use, and sexual experimentation in social settings (49, 54); however, our study did not collect the reasons for kissing same-gender partners.

There are several limitations to our study. First, our study was conducted among heterosexuals attending a public urban sexual health clinic. It may not be generalizable to the general population and other settings where the sexual risks are lower. Our study had a low participation rate, which may cause systematic bias if individuals who responded to the survey were systematically different from individuals who did not. Second, we found some differences in kissing time with the most recent kissing partners when the individual had a regular partner or a casual partner in the past 3 months. However, we did not specifically ask the individuals whether their most recent kissing partner was their regular or casual partner. Third, we could not investigate the association between oropharyngeal gonorrhea and kissing duration or body position because we do not test heterosexuals routinely for oropharyngeal gonorrhea, and it is not recommended in Australia. Fourth, untreated oropharyngeal STIs can last for more 3 months (55, 56), but we did not collect data on kissing partners of more than 3 months prior to the survey. Therefore, caution should be taken when interpreting the association between the duration of kissing and the transmission of STIs. Fifth, our study did not assess the risk of oropharyngeal STIs from activities other than kissing, including fellatio and cunnilingus. Our study was interested in exploring kissing in detail andassessing risk of potential oropharyngeal-oropharyngeal transmission for STIs. Future studies would benefit from separately measuring the contribution of oral sex practices to the transmission of oropharyngeal gonorrhea among heterosexual men and women. Sixth, the duration of kissing was self-reported, and this may have been subjected to recall bias.

## Conclusion

We found that men spent longer than women on top of their most recent opposite-gender kissing partner. Given the mounting evidence in several observational studies from different continents that support kissing as a potential transmission route (19, 20, 57), further research should investigate whether body positioning and duration of kissing influence the risk of gonorrhea transmission. Findings from such studies can inform public health messages regarding the transmission of oropharyngeal STIs.

## Data availability statement

The datasets presented cannot be readily available due to a required policy to protect patient privacy as per the approved ethics. Requests to access the datasets should be directed to JT; JTran@mshc.org.au.

## Ethics statement

The studies involving human participants were reviewed and approved by the Alfred Hospital Ethics Committee, Melbourne, Australia. Written informed consent for participation was not required for this study in accordance with the national legislation and the institutional requirements.

## Author contributions

EC and CF conceived and designed the study with input from JH and provided statistical advice. JT performed data analysis and wrote the first draft of the manuscript. All authors were involved in data interpretation and revising the manuscript for important intellectual content and approved the final version.

## References

[1] Kirkcaldy RD Weston E Segurado AC Hughes G. Epidemiology of gonorrhoea: a global perspective. Sex Health. (2019) 16:401–11. 10.1071/SH19061 31505159 PMC7064409

[2] Chow EPF Grulich AE Fairley CK. Epidemiology and prevention of sexually transmitted infections in men who have sex with men at risk of HIV. Lancet HIV. (2019) 6:e396–405. 10.1016/S2352-3018(19)30043-8 31006612

[3] Whelan J Abbing-Karahagopian V Serino L Unemo M. Gonorrhoea: a systematic review of prevalence reporting globally. BMC Infect Dis. (2021) 21:1152. 10.1186/s12879-021-06381-4 34763670 PMC8582208

[4] Pasipanodya EC Li MJ Jain S Sun X Tobin J Ellorin E. Greater levels of self-reported adherence to pre-exposure prophylaxis (PrEP) are associated with increased condomless sex among men who have sex with men. AIDS Behav. (2020) 24:3192–204. 10.1007/s10461-020-02881-7 32350774 PMC7508761

[5] Gravett RM Westfall AO Overton ET Kudroff K Muzny CA Eaton EF. Sexually transmitted infections and sexual behaviors of men who have sex with men in an American Deep South PrEP clinic. Int J STD AIDS. (2020) 31:127–35. 10.1177/0956462419886228 31992145 PMC7275373

[6] Iniesta C Coll P Barberá MJ García Deltoro M Camino X Fagúndez G. Implementation of pre-exposure prophylaxis programme in Spain. Feasibility of four different delivery models. PLoS ONE. (2021) 16:e0246129. 10.1371/journal.pone.0246129 33556085 PMC7870089

[7] Montaño MA Dombrowski JC Dasgupta S Golden MR Duerr A Manhart LE. Changes in sexual behavior and STI diagnoses among MSM initiating PrEP in a clinic setting. AIDS Behav. (2019) 23:548–55. 10.1007/s10461-018-2252-9 30117076 PMC6368873

[8] Bamberger DM. Trends in sexually transmitted infections. Mo Med. (2020) 117:324–7. 32848268 PMC7431074

[9] Mohammed H Blomquist P Ogaz D Duffell S Furegato M Checchi M. 100 years of STIs in the UK: a review of national surveillance data. Sex Transm Infect. (2018) 94:553–8. 10.1136/sextrans-2017-053273 29654061

[10] Jasek E Chow EP Ong JJ Bradshaw CS Chen MY Hocking JS. Sexually transmitted infections in Melbourne, Australia from 1918 to 2016: nearly a century of data. Commun Dis Intell Q Rep. (2017) 41:E212–22. 29720070 10.33321/cdi.2017.41.31

[11] de Visser RO Badcock PB Rissel C Richters J Smith AMA Grulich AE. Safer sex and condom use: findings from the second Australian study of health and relationships. Sex Health. (2014) 11:495–504. 10.1071/SH14102 25377002

[12] Phillips TR Fairley CK Chen MY Bradshaw CS Chow EPF. Risk factors for urethral gonorrhoea infection among heterosexual males in Melbourne, Australia: 2007–17. Sex Health. (2019) 16:508–13. 10.1071/SH19027 31203836

[13] Fairley CK Hocking JS Zhang L Chow EPF. Frequent transmission of gonorrhea in men who have sex with men. Emerg Infect Dis. (2017) 23:102–4. 10.3201/eid2301.161205 27983487 PMC5176237

[14] Bernstein KT Chesson H Kirkcaldy RD Marcus JL Gift TL Aral SO. Kiss and tell: limited empirical data on oropharyngeal neisseria gonorrhoeae among men who have sex with men and implications for modeling. Sex Transm Dis. (2017) 44:596–8. 10.1097/OLQ.0000000000000709 28910265 PMC5967843

[15] Hook EW 3rd Bernstein K. Kissing, saliva exchange, and transmission of *Neisseria gonorrhoeae*. Lancet Infect Dis. (2019) 19:e367–9. 10.1016/S1473-3099(19)30306-8 31324518 PMC6764880

[16] Fairley CK Cornelisse VJ Hocking JS Chow EPF. Models of gonorrhoea transmission from the mouth and saliva. Lancet Infect Dis. (2019) 19:e360–6. 10.1016/S1473-3099(19)30304-4 31324517

[17] Chow EP Tabrizi SN Phillips S Lee D Bradshaw CS Chen MY. *Neisseria gonorrhoeae* bacterial DNA load in the pharynges and saliva of men who have sex with men. J Clin Microbiol. (2016) 54:2485–90. 10.1128/JCM.01186-16 27413195 PMC5035428

[18] Chow EPF Lee D Tabrizi SN Phillips S Snow A Cook S. Detection of Neisseria gonorrhoeae in the pharynx and saliva: implications for gonorrhoea transmission. Sex Transm Infect. (2016) 92:347. 10.1136/sextrans-2015-052399 26622046

[19] Chow EPF Cornelisse VJ Williamson DA Priest D Hocking JS Bradshaw CS. Kissing may be an important and neglected risk factor for oropharyngeal gonorrhoea: a cross-sectional study in men who have sex with men. Sex Transm Infect. (2019) 95:516–21. 10.1136/sextrans-2018-053896 31073095

[20] Chow EPF Vodstrcil LA Williamson DA Maddaford K Hocking JS Ashcroft M. Incidence and duration of incident oropharyngeal gonorrhoea and chlamydia infections among men who have sex with men: prospective cohort study. Sex Transm Infect. (2021) 97:452–7. 10.1136/sextrans-2020-054764 33208509

[21] Hook EW 3rd Handsfield HH. Gonococcal Infections in the adult. In: Holmes KK Sparling PK Stamm WE Piot P Wasserheit JN Corey L, editors. Sex Transm Dis. 4th ed. New York, NY: McGraw-Hill Medical (2008). p. 627–46.

[22] Bro-Jorgensen A Jensen T. Gonococcal pharyngeal infections. Report of 110 cases. Br J Vener Dis. (1973) 49:491–9. 10.1136/sti.49.6.491 4202719 PMC1048392

[23] Sackel SG Alpert S Fiumara NJ Donner A Laughlin CA McCormack WM. Orogenital contact and the isolation of *Neisseria gonorrhoeae, Mycoplasma hominis*, and *Ureaplasma urealyticum* from the pharynx. Sex Transm Dis. (1979) 6:64–8. 10.1097/00007435-197904000-00004 115097

[24] Allen C Fairley CK Chen MY Maddaford K Ong JJ Williamson DA. Oropharyngeal gonorrhoea infections among heterosexual women and heterosexual men with urogenital gonorrhoea attending a sexual health clinic in Melbourne, Australia. Clin Microbiol Infect. (2021) 27:1799–804. 10.1016/j.cmi.2021.03.033 33845205

[25] Chow EPF Chen MY Williamson DA Bradshaw CS Vodstrcil LA Trumpour S. Oropharyngeal and genital gonorrhea infections among women and heterosexual men reporting sexual contact with partners with gonorrhea: implication for oropharyngeal testing of heterosexual gonorrhea contacts. Sex Transm Dis. (2019) 46:743–7. 10.1097/OLQ.0000000000001068 31517767

[26] Read PJ Wand H Guy R Donovan B McNulty AM. Unprotected fellatio between female sex workers and their clients in Sydney, Australia. Sex Transm Infect. (2012) 88:581–4. 10.1136/sextrans-2011-050430 22875839

[27] Sacomori C Cardoso FL. Sexual initiative and intercourse behavior during pregnancy among brazilian women: a retrospective study. J Sex Marital Ther. (2010) 36:124–36. 10.1080/00926230903554503 20169493

[28] Herbenick D Fu TJ Owens C Bartelt E Dodge B Reece M. Kissing, cuddling, and massage at most recent sexual event: findings from a U. S nationally representative probability sample. J Sex Marital Ther. (2019) 45:159–72. 10.1080/0092623X.2018.1494648 30040548

[29] Hughes SM Harrison MA Gallup GG. Sex differences in romantic kissing among college students: an evolutionary perspective. Evol Psychol. (2007). 10.1177/147470490700500310

[30] Lefkowitz ES Wesche R Leavitt CE. Never been kissed: correlates of lifetime kissing status in U. S university students. Arch Sex Behav. (2018) 47:1283–93. 10.1007/s10508-018-1166-y 29464454 PMC5893370

[31] Lefkowitz ES Wesche R Picci G Hochgraf AK. Daily associations between kissing and affect during the transition from adolescence to young adulthood. J Res Adolesc. (2018) 28:779–85. 10.1111/jora.12422 29927006

[32] Charleson FJ Fairley CK Hocking JS Vodstrcil LA Bradshaw CS Chow EPF. Age, ethnic and travel-related disparities in kissing and sexual practices among heterosexual men in Melbourne, Australia. Sex Health. (2020) 17:279–87. 10.1071/SH19230 32571477

[33] Moore EA Kulibert D Thompson AE. Is a kiss just a kiss? predicting variations in motives for romantic kissing. J Relatsh Res. (2017) 8:e3. 10.1017/jrr.2017.4

[34] Welsh DP Haugen PT Widman L Darling N Grello CM. Kissing is good: A developmental investigation of sexuality in adolescent romantic couples. Sex Res Soc Policy. (2005) 2:32–41. 10.1525/srsp.2005.2.4.32

[35] Chow EPF Hocking JS Ong JJ Phillips TR Schmidt T Buchanan A. Brief report: changes in PrEP use, sexual practice, and use of face mask during sex among MSM during the second wave of COVID-19 in Melbourne, Australia. J Acquir Immune Defic Syndr. (2021) 86:153–6. 10.1097/QAI.0000000000002575 33433122 PMC7808277

[36] Chow EPF Hocking JS Ong JJ Phillips TR Fairley CK. Sexually transmitted infection diagnoses and access to a sexual health service before and after the national lockdown for COVID-19 in Melbourne, Australia. Open Forum Infect Dis. (2021) 8:ofaa536. 10.1093/ofid/ofaa536 33506064 PMC7665697

[37] Chow EPF Carlin JB Read TRH Chen MY Bradshaw CS Sze JK. Factors associated with declining to report the number of sexual partners using computer-assisted self-interviewing: a cross-sectional study among individuals attending a sexual health centre in Melbourne, Australia. Sex Health. (2018) 15:350–7. 10.1071/SH18024 29966584

[38] Vodstrcil LA Hocking JS Cummings R Chen MY Bradshaw CS Read TRH. Computer assisted self interviewing in a sexual health clinic as part of routine clinical care; impact on service and patient and clinician views. PLoS ONE. (2011) 6:e18456. 10.1371/journal.pone.0018456 21483799 PMC3069102

[39] Maldonado G Greenland S. Simulation study of confounder-selection strategies. Am J Epidemiol. (1993) 138:923–36. 10.1093/oxfordjournals.aje.a116813 8256780

[40] Mandal S Wu HM MacNeil JR Machesky K Garcia J Plikaytis BD. Prolonged university outbreak of meningococcal disease associated with a serogroup B strain rarely seen in the United States. Clin Infect Dis. (2013) 57:344–8. 10.1093/cid/cit243 23595832

[41] McMillan M Walters L Mark T Lawrence A Leong LEX Sullivan T. B Part of It study: a longitudinal study to assess carriage of Neisseria meningitidis in first year university students in South Australia. Hum Vaccin Immunother. (2019) 15:987–94. 10.1080/21645515.2018.1551672 30513251 PMC6605849

[42] Watle SV Caugant DA Tunheim G Bekkevold T Laake I Brynildsrud OB. Meningococcal carriage in Norwegian teenagers: strain characterisation and assessment of risk factors. Epidemiol Infect. (2020) 148:e80. 10.1017/S0950268820000734 32228726 PMC7189347

[43] MacLennan J Kafatos G Neal K Andrews N Cameron JC Roberts R. Social behavior and meningococcal carriage in British teenagers. Emerg Infect Dis. (2006) 12:950–7. 10.3201/eid1206.051297 16707051 PMC3373034

[44] Grimm JM Schmeling DO Dunmire SK Knight JA Mullan BD Ed JA. Prospective studies of infectious mononucleosis in university students. Clin Transl Immunology. (2016) 5:e94. 10.1038/cti.2016.48 27588199 PMC5007628

[45] Crawford DH Macsween KF Higgins CD Thomas R McAulay K Williams H. A cohort study among university students: identification of risk factors for Epstein-Barr virus seroconversion and infectious mononucleosis. Clin Infect Dis. (2006) 43:276–82. 10.1086/505400 16804839

[46] Ngai S Weiss D Bell JA Majrud D Zayas G Crawley A. Carriage of neisseria meningitidis in men who have sex with men presenting to public sexual health clinics, New York City. Sex Transm Dis. (2020) 47:541–8. 10.1097/OLQ.0000000000001205 32520884

[47] Odegaard K. Kissing as a mode of transmission of infectious mononucleosis. Lancet. (1967) 1:1052–3. 10.1016/S0140-6736(67)91559-0 4164481

[48] Crawford DH Swerdlow AJ Higgins C McAulay K Harrison N Williams H. Sexual history and epstein-barr virus infection. J Infect Dis. (2002) 186:731–6. 10.1086/342596 12198605

[49] Priest D Chow EPF. Kissing while high on ecstasy: lessons from a gay dance party attendee. Sex Transm Infect. (2018) 94:143. 10.1136/sextrans-2017-053427 29463767

[50] Chow EPF Vodstrcil LA Fairley CK. Seasonal variations in kissing and sexual activities among men who have sex with men in Melbourne, Australia: implications for seasonal sexually transmissible infection preventions and interventions. Sex Health. (2020) 17:149–54. 10.1071/SH19046 32135076

[51] Cornelisse VJ Priest D Fairley CK Walker S Bradshaw CS Phillips T. The frequency of kissing as part of sexual activity differs depending on how men meet their male casual sexual partners. Int J STD AIDS. (2018) 29:598–602. 10.1177/0956462417748717 29256822

[52] Chow EP Phillips TR Tran J Maddaford K Fairley CK. A cross-sectional study of male and female kissing partners among men who have sex with men. Sexual Health. (2022) 19:27–32. 10.1071/SH21184 35241217

[53] Kilner A Fairley CK Burrell S Bradshaw CS Chen MY Chow EPF. Age pattern of sexual activities with the most recent partner among men who have sex with men in Melbourne, Australia: a cross-sectional study. BMJ Sex Reprod Health. (2021) 47:e4. 10.1136/bmjsrh-2020-200720 32868336

[54] Yost MR McCarthy L. Girls Gone Wild? Heterosexual women's same-sex encounters at college parties. Psychol Women Q. (2011) 36:7–24. 10.1177/0361684311414818

[55] Chow EP Camilleri S Ward C Huffam S Chen MY Bradshaw CS. Duration of gonorrhoea and chlamydia infection at the pharynx and rectum among men who have sex with men: a systematic review. Sex Health. (2016) 13:199–204. 10.1071/SH15175 26886136

[56] Chow EP Fairley CK. The role of saliva in gonorrhoea and chlamydia transmission to extragenital sites among men who have sex with men: new insights into transmission. J Int AIDS Soc. (2019) 22:e25354. 10.1002/jia2.25354 31468730 PMC6715946

[57] Barbee LA Soge OO Khosropour CM Haglund M Yeung W Hughes J. The duration of pharyngeal gonorrhea: a natural history study. Clin Infect Dis. (2021) 73:575–82. 10.1093/cid/ciab07 33513222 PMC8366826

