# Supplementary material for: The duration and body position during tongue-kissing among heterosexual men and women
Source: Front Public Health. 2022 Dec 22;10:934962. doi: 10.3389/fpubh.2022.934962 (PMC9814118; doi:10.3389/fpubh.2022.934962)
Supplement: Supplementary file 1 [file Data_Sheet_1.docx]

**Table S1.**  Univariable and multivariable linear regression analyses of the association between demographic factors and the duration of kissing among 2514 heterosexual men and women who only had opposite-gender kissing partners in the past three months

| **Predictors** | **Crude regression coefficient**  **(95% CI)** | **Unadjusted**  **mean**  **(95% CI)** | **P-value** | **Adjusted regression coefficient**  **(95% CI)** | **Adjusted mean**  **(95% CI)** | **P value** |
| --- | --- | --- | --- | --- | --- | --- |
| **Gender** |  |  |  |  |  |  |
| *Female* | 1 | 11.02 (10.25, 11.79) | Ref | 1 | 11.34 (10.58, 12.09) | Ref |
| *Male* | 1.28 (0.21, 2.35) | 12.31 (11.57, 13.05) | 0.019 | 0.68 (-0.38, 1.73) | 12.01 (11.29, 12.74) | 0.210 |
| **Age (years)** | -0.02 (-0.08, 0.04) |  | 0.562 |  |  |  |
| **Country of birth** |  |  |  |  |  |  |
| *Australia* | 1 | 14.40 (13.51, 15.30) | Ref |  | 14.39 (13.50, 15.28) | Ref |
| *Overseas* | -4.29 (-5.42, -3.17) | 10.11 (9.43, 10.79) | <0.001 | -4.2 (-5.38, -3.13) | 10.13 (9.46, 10.81) | <0.001 |
| *Unknown* | -2.47 (-4.92, -0.03) | 11.93 (9.66, 14.21) | 0.048 | -2.62 (-5.04, -0.20) | 11.78 (9.52, 14.02) | 0.034 |
| **Regular sex partners** |  |  |  |  |  |  |
| *No* | 1 | 13.18 (12.44, 13.91) | Ref |  | 12.61 (11.85, 13.37) | Ref |
| *Yes* | -3.17 (-4.25, -2.09) | 10.00 (9.21, 10.79) | <0.001 | -2.07 (-3.24, -0.91) | 10.54 (11.85, 13.37) | 0.001 |
| *Unknown* | -2.19 (-5.45, 1.10) | 10.99 (7.81, 14.16) | 0.187 | 0.25 (-3.13, 3.63) | 12.86 (9.62, 16.11) | 0.884 |
| **Casual sex partners** |  |  |  |  |  |  |
| *No* | 1 | 9.05 (7.62, 10.47) | Ref |  | 9.74 (8.27, 11.20) | Ref |
| *Yes* | 3.71 (2.15, 5.26) | 12.75 (12.14, 13.37) | <0.001 | 2.80 (1.17, 4.42) | 12.54 (11.91, 13.16) | 0.001 |
| *Unknown* | -0.97 (-3.06, 1.12) | 8.08 (6.55, 9.61) | 0.0363 | -1.08 (-3.20, 1.03) | 8.65 (7.04, 10.27 | 0.315 |

CI, confidence interval

| **Predictors** | **Crude regression coefficient**  **(95% CI)** | **Unadjusted**  **mean (95% CI)** | **P-value** | **Adjusted regression coefficient**  **(95% CI)** | **Adjusted means (95% CI)** | **P value** |
| --- | --- | --- | --- | --- | --- | --- |
| **Gender** |  |  |  |  |  |  |
| *Female* | 1 | 7.19 (6.72, 7.66) | Ref | 1 | 7.37 (6.90, 7.84) | Ref |
| *Male* | 1.13 (0.49, 1.77) | 8.32 (7.88, 8.76) | 0.001 | 0.79 (0.15,1.44) | 8.17 (7.73, 8.60) | 0.016 |
| **Age (years)** | -0.01 (-0.05, 0.03) |  | 0.587 |  |  |  |
| **Country of birth** |  |  |  |  |  |  |
| *Australia* | 1 | 9.13 (8.60, 9.67) | Ref | 1 | 9.06 (8.52, 9.60) | Ref |
| *Overseas* | -2.14 (-2.82, -1.46) | 6.99 (6.58, 7.41) | <0.001 | -2.02 (-2.71, -1.34) | 7.04 (6.62, 7.45) | <0.001 |
| *Unknown* | -1.23 (-2.71, 0.25) | 7.90 (6.52, 9.28) | 0.104 | -1.18 (-2.66, 0.29) | 7.88 (6.51, 9.25) | 0.116 |
| **Regular sex partners** |  |  |  |  |  |  |
| *No* | 1 | 8.44 (7.99, 8.89) | Ref |  | 8.22 (7.75, 8.70) | Ref |
| *Yes* | -1.36 (-2.02, -.7135693) | 7.08 (6.61, 7.55 | <0.001 | -0.95 (-1.66, -0.24) | 7.27 (6.79, 7.76) | 0.009 |
| *Unknown* | -0.44 (-2.53, 1.65) | 8 (5.96, 10.04) | 0.678 | 0.55 (-1.61, 2.71) | 8.77 (6.69, 10.85) | 0.618 |
| **Casual sex partners** |  |  |  |  |  |  |
| *No* | 1 | 6.88(6.02, 7.74) | Ref | 1 | 7.28 (6.39, 8.17) | Ref |
| *Yes* | 1.31 (0.38, 2.25) | 8.20 (7.83, 8.57) | 0.006 | 0.82 (-.18, 1.81) | 8.09 (7.71, 8.47) | 0.107 |
| *Unknown* | -0.61 (-1.89, 0.67) | 6.28 (5.33, 7.22) | 0.353 | -0.80 (-2.09, 0.49) | 6.47 (5.49, 7.47) | 0.223 |

**Table S2.** Univariable and multivariable linear regression analyses of the association between demographic factors and the duration of kissing while on top of partner among 2145 heterosexual men and women who only had opposite-gender kissing partners in the past three months

CI, confidence interval

| **Predictors** | **Crude regression coefficient**  **(95% CI)** | **Unadjusted**  **Mean (95% CI)** | **P-value** | **Adjusted regression coefficient**  **(95% CI)** | **Adjusted mean**  **(95% CI)** | **P value** |
| --- | --- | --- | --- | --- | --- | --- |
| **Gender** |  |  |  |  |  |  |
| *Female* | 1 | 8.24 (7.76, 8.71) | Ref | 1 | 8.39(7.92, 8.86) | Ref |
| *Male* | -0.64 (-1.30, 0.03) | 7.60 (7.13, 8.07) | 0.061 | -0.94 (-1.61, -0.28) | 7.45 ( 6.98, 7.91) | 0.006 |
| **Age (years)** | -0.02 (-0.06, 0.02) |  | 0.378 |  |  |  |
| **Country of birth** |  |  |  |  |  |  |
| *Australia* | 1 | 9.08 ( 8.52, 9.64) | Ref | 1 | 9.20 (8.64, 9.76) | Ref |
| *Overseas* | -1.84 (-2.55, -1.14) | 7.24 (6.81, 7.67) | <0.001 | -2.03 (-2.74, -1.32) | 7.17 ( 6.74, 7.59) | <0.001 |
| *Unknown* | -1.22 (-2.76, 0.32) | 7.86 (6.42, 9.30) | 0.121 | -1.27 (-2.81, 0.26) | 7.93 (6.50, 9.35) | 0.103 |
| **Regular sex partners** |  |  |  |  |  |  |
| *No* | 1 | 8.76 ( 8.29, 9.22) | Ref | 1 | 8.65 (8.16, 9.13) | Ref |
| *Yes* | -1.78 (-2.46, -1.11) | 6.97 ( 6.49, 7.46) | <0.001 | -1.58 (-2.32, -0.85) | 7.06 (6.56, 7.57) | <0.001 |
| *Unknown* | -0.64 (-2.83, 1.56) | 8.12 (5.98, 10.27) | 0.570 | 0.01 (-2.26, 2.26) | 8.65 (6.46, 10.84) | 0.999 |
| **Casual sex partners** |  |  |  |  |  |  |
| *No* | 1 | 7.491 (6.58, 8.40) | Ref | 1 | 8.07 (7.12, 9.01) | Ref |
| *Yes* | 0.75 (-0.24, 1.73) | 8.24 (7.86, 8.62) | 0.138 | 0.01 (-1.04, 1.04) | 8.07 (7.68, 8.46) | 0.999 |
| *Unknown* | -1.28 (-2.62, 0.07) | 6.22 (5.22, 7.21) | 0.063 | -1.39 (-2.75, -0.04) | 6.68 (5.63, 7.72) | 0.044 |

**Table S3.** Univariable and multivariable linear regression analyses of the association between demographic factors and the duration of kissing while lying down underneath partner among 2055 heterosexual men and women who only had opposite-gender kissing partners in the past three months

CI, confidence interval
