# Supplementary material for: The duration and body position during tongue-kissing among heterosexual men and women
Source: Front Public Health. 2022 Dec 22;10:934962. doi: 10.3389/fpubh.2022.934962 (PMC9814118; doi:10.3389/fpubh.2022.934962)

Figure S1. Visual representation of kissing while on top of and lying down underneath an opposite-gender kissing partner

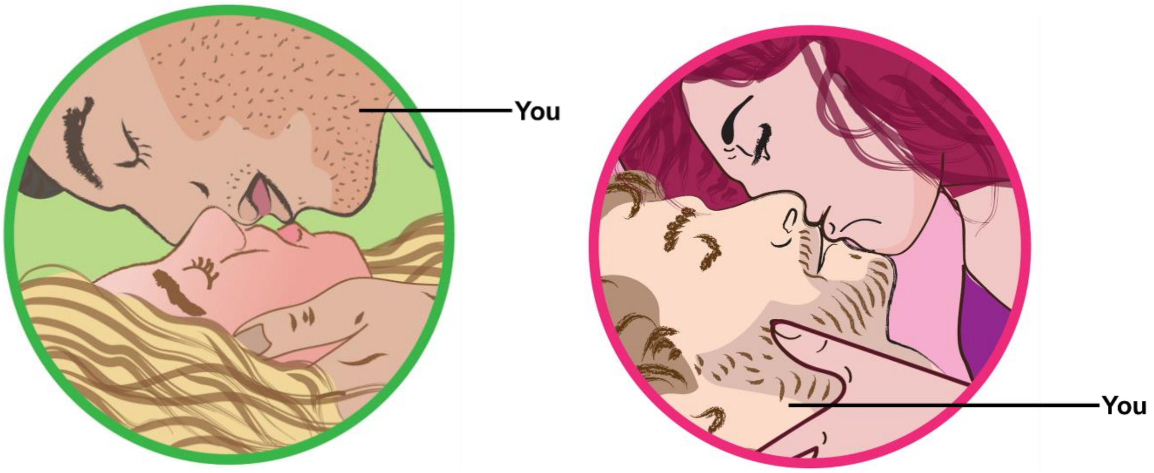

Supplement: Supplementary file 2 [file Image_1.pdf]
